# Central corticotropin releasing factor and social stress

**DOI:** 10.3389/fnins.2013.00117

**Published:** 2013-07-09

**Authors:** Tobias Backström, Svante Winberg

**Affiliations:** ^1^Department of Wildlife, Fish, and Environmental Studies, Swedish University of Agricultural SciencesUmeå, Sweden; ^2^Department of Neuroscience, Uppsala UniversityUppsala, Sweden

**Keywords:** corticotropin releasing factor (CRF), CRF-receptors, dominance hierarchies, hypothalamic–pituitary–adrenal axis, social defeat, social isolation, social stress

## Abstract

Social interactions are a main source of stress in vertebrates. Social stressors, as well as other stressors, activate the hypothalamic–pituitary–adrenal (HPA) axis resulting in glucocorticoid release. One of the main components of the HPA axis is corticotropin releasing factor (CRF). The neuropeptide CRF is part of a peptide family including CRF, urocortin 1–3, urotensin 1–3, and sauvagine. The actions of the CRF family are mediated by at least two different receptors with different anatomical distribution and affinities for the peptides. The CRF peptides affect several behavioral and physiological responses to stress including aggression, feeding, and locomotor activity. This review will summarize recent research in vertebrates concerning how social stress interacts with components of the CRF system. Consideration will be taken to the different models used for social stress ranging from social isolation, dyadic interactions, to group dominance hierarchies. Further, the temporal effect of social stressor from acute, intermittent, to chronic will be considered. Finally, strains selected for specific behavior or physiology linked to social stress will also be discussed.

## Introduction

**Figure 1:**
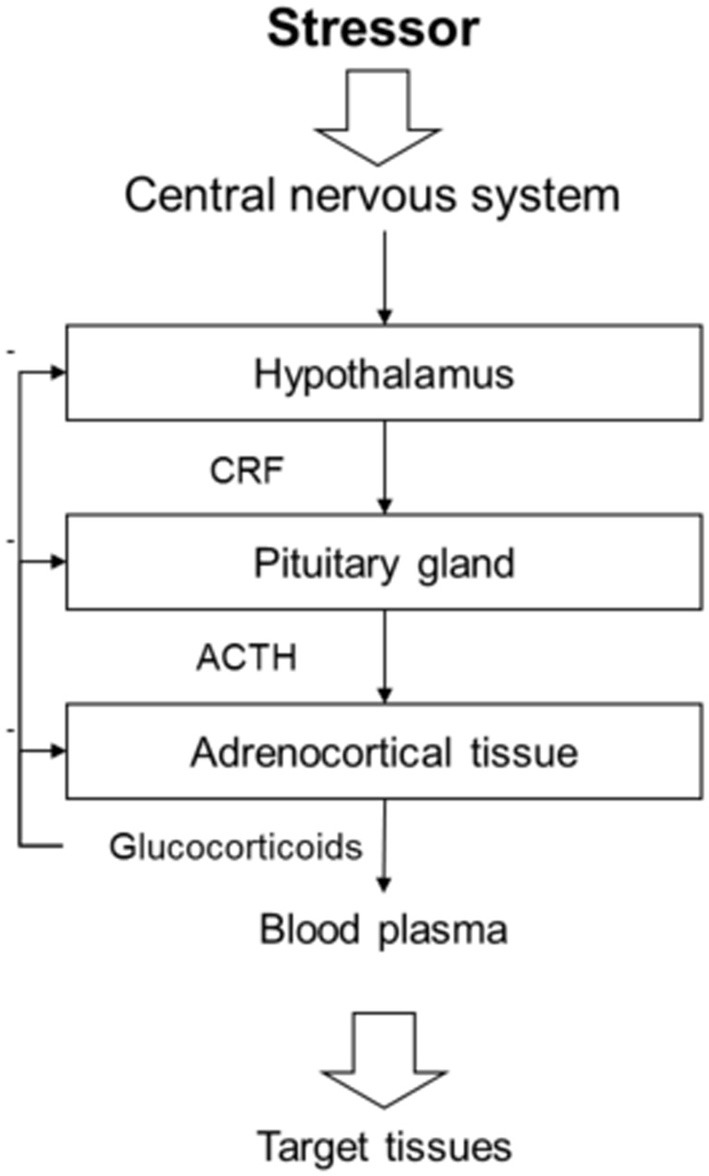
**Schematic drawing of the hypothalamic pituitary adrenocortical axis.** The primary physiological stress response is partly mediated by the hypothalamic–pituitary–adrenocortical (HPA) axis in mammals. A stressor activates the central nervous system and the hypothalamus then releases corticotropin releasing factor (CRF) unto the pituitary gland. The pituitary gland will in its turn release adrenocorticotropic hormone (ACTH), which induces glucocorticoid synthesis and release from the adrenal tissue into the blood. The glucocorticoids will then affect target tissues throughout the body mainly to increase energy availability and inhibit processes not necessary for the immediate survival. Each step of the HPA axis is self-regulated by an array of feedback loops.

The stress response elicited by social stressors does not differ from the response to other challenges. A stressor, which can be a real or perceived threat, causes a physiological response aimed at counteracting a homeostatic disruption. The immediate effect of the stress response is to prepare the animal for a quick and energetic reaction, often referred to as “the fight-or-flight response” (Cannon, 1915). “The fight-or-flight response” involves many different adaptations mainly by increasing energy availability and inhibiting processes not necessary for the immediate survival (Johnson et al., 1992; Carrasco and Van De Kar, 2003). The primary physiological stress response is mediated by the sympathetic nervous system and hypothalamic–pituitary–adrenal (HPA) axis in mammals (see Figure 1) and the hypothalamic–pituitary–interrenal (HPI) axis in teleost fish (Wendelaar Bonga, 1997; Carrasco and Van De Kar, 2003). During a stress response, the hypothalamus is activated and releases corticotropin releasing factor (CRF; also known as corticotropin releasing hormone, CRH), which stimulates the release of adrenocorticotropic hormone (ACTH) from the pituitary gland. ACTH induces glucocorticoid synthesis and release from the adrenal/interrenal tissue into the blood. The HPA/HPI axis is self-regulated by an array of feedback loops (Carrasco and Van De Kar, 2003).

One of the main mediators of the stress response is CRF and here we will briefly detail the distribution and main effectors of the CRF system [for a more detailed description, see review by Ronan and Summers (2011)]. CRF was initially discovered in 1955 when factors in the hypothalamus were found to induce ACTH release from the pituitary gland (Guillemin and Rosenberg, 1955; Saffran and Schally, 1955). In 1981, CRF was characterized as a neuropeptide of 41 amino acids (Vale et al., 1981) inducing ACTH release from the pituitary gland (Lederis et al., 1985). Cells synthesizing CRF are primarily found in the hypothalamus, more precisely in the parvocellular neurons of the paraventricular nucleus (PVN) in mammals (Bloom et al., 1982; Pelletier et al., 1983; Swanson et al., 1983; Raadsheer et al., 1993). CRF is also found in other brain areas including the magnocellular cells of the PVN and supraoptic nucleus (Paull and Gibbs, 1983; Delville et al., 1992; Luo et al., 1994). The CRF system seems to be well conserved since CRF synthesizing cells are found in the parvocellular neurons in the preoptic area (POA) in teleost fish (Matz and Hofeldt, 1999; Pepels et al., 2002), the teleostean POA being homologues to the mammalian PVN. A recent study suggests that CRF is also present in other areas in the teleost brain, outside the POA such as the dorsal telencephalon and suprachiasmatic nucleus (Alderman and Bernier, 2007).

The effects of CRF are mediated by at least two different receptor types, namely corticotropin releasing factor receptor 1 (CRF-R1) and corticotropin releasing factor receptor 2 (CRF-R2), and these appear to be present in both mammals (Chang et al., 1993; Chen et al., 1993; Perrin et al., 1993, 1995; Vita et al., 1993; Kishimoto et al., 1995; Lovenberg et al., 1995; Stenzel et al., 1995) and teleost fish (Arai et al., 2001; Pohl et al., 2001). The CRF-R1 have been reported to mediate the activation of the HPA/HPI axis (Timpl et al., 1998; Bale et al., 2002b; Huising et al., 2004) and to induce anxiety-like behavior (Britton et al., 1986; Heinrichs et al., 1997; Bale et al., 2002b), whereas the CRF-R2 have been reported to affect several other behavioral and physiological responses to stress (Liebsch et al., 1999; Coste et al., 2000; Bale et al., 2002b), and also to be involved in anxiety control (Bale et al., 2000, 2002a). In addition to the receptors, there is a CRF binding protein (CRF-BP), which has been reported in mammals (Potter et al., 1991; Cortright et al., 1995; Behan et al., 1996) and the teleost Mozambique tilapia (*Tilapia mossambicus*) (Seasholtz et al., 2002). The CRF-BP has been suggested to bind CRF with high affinity and thereby decrease CRF effects (Potter et al., 1991; Woods et al., 1994; Cortright et al., 1995).

Several neuropeptides closely related to CRF have been identified including urocortin-1, urocortin-2, and urocortin-3 in mammals (Vaughan et al., 1995; Lewis et al., 2001; Reyes et al., 2001), sauvagine in anurans (Montecucchi et al., 1979), and urotensin-I (UI) in teleost fish (Lederis et al., 1982; Ishida et al., 1986; Bernier et al., 1999). The CRF-R1 binds both CRF and urocortin 1 with similar affinity whereas the CRF-R2 has a higher affinity for urocortin-1 (Vaughan et al., 1995; Donaldson et al., 1996; Ozawa et al., 1998). Urocortin-2, urocortin-3, UI, and sauvagine are mainly bound by CRF-R2 (Hauger et al., 2003). Thus, CRF-R1 main ligands are CRF and urocortin-1, whereas CRF-R2 main ligands are urocortin-2, urocortin-3, UI, and sauvagine.

Several behaviors in mammals are affected by CRF, and CRF has been reported to increase locomotor activity, elevate anxiety-like responses, and reduce feeding in vertebrates [reviewed by Heinrichs and Koob (2004) and Lowry and Moore (2006)]. Further, the CRF system is an important mediator of several behavioral stress responses [see reviews by Bale and Vale (2004), Heinrichs and Koob (2004), and Lowry and Moore (2006)]. These and several other excellent reviews describe how CRF, behavior, and physiology are interacting. However, the specifics of how social stressors and the CRF system interact have not been reviewed. Therefore, the focus of this review is on how social stressors and CRF are interacting in modulating behavior and physiology.

## Social stressors

Social systems and social stressors differ between species (Blanchard et al., 2001), but generally social animals form dominance-based social hierarchies. In these hierarchies, each individual has a social rank position. For example, dominance hierarchies can be linear, which means that the alpha individual, having the highest rank, dominates all other group members, whereas the beta individual being subordinate to the alpha individual dominates the rest of the group members, and so on until the omega individual, which has the lowest rank and is subordinate to all group members (Huntingford and Turner, 1987). Dominance hierarchies are often formed by agonistic interactions. These agonistic interactions are performed by two or more individuals competing for the higher social position. Initially, a phase of mutual displays and threats escalates into a phase of overt aggressive behavior including violent attacks (Huntingford and Turner, 1987). The overt aggressive behavior continues until inter-relational rank is firmly established and the subordinate individuals signal defeat and retreat. However, even after the hierarchy is established subordinate animals are subjected to social stress and show signs of chronic stress (Greenberg et al., 1984; Sapolsky, 1990; Winberg et al., 1991; Blanchard et al., 1993), especially in captivity or other situations where escape is not possible. Experimentally, the effect of social stress is often studied using dyads and thus creating one dominant and one subordinate individual. Similar pairing is also used for the resident–intruder test, where the resident typically is an experienced and aggressive fighter and thus predestined to become dominant. Intruders from these pairings are suffering social defeat, which is another common social stress model.

Social isolation can be stressful as well in several vertebrates, usually depending on social organization. In several species, either isolation or grouping would be stressful and some gender differences could also be apparent (Blanchard et al., 2001). Rodent models are often used for both social isolation and dominance hierarchies, whereas teleost fish has mainly been used in dominance hierarchical studies such as dyadic interactions. Several experiments have shown that the CRF system is important in mediating effects of social stress on aggression and anxiety-like behaviors. Early studies were mainly done using exogenous administration of CRF and have continued with specific antagonist as well as site-specific administration. Further studies have been using the expression of CRF mRNA, as well as the expression of other genes related to the CRF system, to monitor the effects of acute and social stressors. Recent studies have also been using genetic engineering and thus creating deficient or knockout strains for elucidating the effects of various components of the CRF system. In the following sections, these studies will be reviewed.

## Acute effects of exogenous CRF

Aggressive behavior is crucial for establishing dominance, upholding territories, and protecting offspring. Several studies using exogenous CRF have reported modulating effects on various types of aggressive behavior in various vertebrates. However, the function of the CRF system in the control of aggressive behavior is not consistent across species. It seems that it can both induce and reduce aggression. For instance, in male mice intracerebroventricular (icv) injection of CRF as well as sauvagine reduces aggression (Mele et al., 1987). Similarly, in female mice icv injections of either CRF, urocortin-1, or urocortin-3 inhibit maternal aggression (Gammie et al., 2004; D'anna et al., 2005). Further, these responses seems to be mediated by CRF-R1, since the specific CRF-R1 antagonist antalarmin injected into the amygdala reduces defensive posture in socially defeated mice (Robison et al., 2004). In contrast, in male rats injections of CRF into the amygdala can increase aggression (Elkabir et al., 1990). Further, icv injections of the CRF non-selective antagonist α-helical CRH_1−41_ decrease aggressive behavior in male rats (Aloisi et al., 1999). Similarly, the CRF-R1 antagonist SSR125543A delayed the latency to attack intruders in Syrian hamsters (*Mesocricetus auratus*) (Farrokhi et al., 2004). It also seems as if CRF-R antagonists reduce the behavioral effects of aggression and defeat as a social stressor. For instance, the CRF-R1 antagonist NBI-30775 increased time in defensive posture and latency to submission, and reduced the defeat stress induced immobility in rats (Wood et al., 2012). Further, the CRF non-selective antagonist d-Phe CRF_(12−41)_ injected into the dorsal raphe nucleus of Syrian hamster reduced both the acquisition and expression of conditioned defeat as seen by reduced submissive and defensive behavior, whereas the CRF-R2 specific antagonist anti-Svg-30 only reduced the expression of conditioned defeat (Cooper and Huhman, 2007). In a follow-up experiment, it was found that an injection of a non-selective CRF antagonist into the lateral ventricle reduced submissive and defensive behavior, and similarly a selective CRF-R2 antagonist also reduced submissive and defensive behavior, but a CRF-R1 antagonist had no effect on these behaviors (Cooper and Huhman, 2010). Thus, there is evidence for the involvement of both CRF-R1 and CRF-R2 in modulation of aggressive behavior. However, since CRF by itself elicits divergent responses in aggressive behavior, species-specific patterns could be present.

Interestingly, this ambiguity concerning the effect of CRF on aggression can be seen in a single species of teleost fish. In rainbow trout (*Onchorhynchus mykiss*), two studies using exogenous CRF have reached two different conclusions. Carpenter et al. (2009) found that an icv injection of CRF increased victory chances in dyadic interactions and decreased latency to attack in winners but also reduced aggressive interactions. However, Backström et al. (2011a) found, using similar injection and doses, that CRF induced subordinance during dyadic interactions but saw no effect on number of attacks or latency to attack. However, the longevity of fights might be involved since Carpenter et al. used 15 min and Backström et al. used 60 min of social interactions. Further, no modulating effects on aggression could be seen either by UI, the CRF-R antagonists α-helical CRH_1−41_, or antalarmin (Backström et al., 2011a).

Studies applying administration of exogenous CRF have also shown that CRF is involved in anxiety-like behavior during social interactions (Arregi et al., 2006). In an early study on rats, icv injection of CRF reduced the number of social interactions indicating an anxiogenic effect (Dunn and File, 1987). Anxiety-like behavior in socially defeated male rats is reduced by injection of antisense oligodeoxynucleotide for CRF-R1 (Liebsch et al., 1995). Similarly, CRF and sauvagine reduces sociability in male mice (Mele et al., 1987). Urocortin-2 intraperitoneally injected is anxiogenic in prairie voles (*Microtus ochrogaster*) as indicated by increasing spontaneous parental behavior following injection (Samuel et al., 2008). In addition, icv administration of urocortin-3 as well as the CRF-R1 agonist, stressin_1_-A, in rats also induces anxiety-like behavior (Zhao et al., 2007). However, the sodium lactate-induced panic-like behavior in male rats during social interaction test can be blocked by the CRF-R1 antagonist antalarmin injected intraperitoneally (Shekhar et al., 2011). Further, injections of CRF and urocortin-1 into the basolateral amygdala reduces social interactions and thus indicating an anxiety-like behavior (Rainnie et al., 2004; Spiga et al., 2006), and the CRF-R1 specific antagonist NBI3b1996 injected into the basolateral nucleus of the amygdala attenuates the anxiety-like behavior (Gehlert et al., 2005). Single and repeated injections of urocortin-1 into the bed nucleus of the stria terminalis induce anxiety-like behavior during social interaction test in male rats (Lee et al., 2008), indicating a difference in function between basolateral amygdala and bed nucleus of the stria terminalis. Thus, there is evidence of involvement of both CRF-R1 and CRF-R2 in anxiety-like behavior in mammals, but CRF-R1 seems to be most important for this effect. It seems as if the anxiety-like behavior expressed in vertebrates is well conserved since it has been reported in teleost fish as well. In rainbow trout, icv injections of CRF or UI have been shown to induce an anxiety-like behavior similar to a non-ambulatory motor activity in rodents both in isolation and in dyadic interactions (Carpenter et al., 2007, 2009; Backström et al., 2011a). However, no effects of CRF antagonists could be seen, including antalarmin, which has been shown to reduce avoidance behavior in crucian carp (*Carassius carassius*) (Lastein et al., 2008).

## Intermittent effects of CRF—expression studies

Social stressors affect gene expression in the CRF system. Because of the involvement in the HPA-axis an up-regulation of CRF expression would be expected. This can also be seen in several studies using social isolation. For instance, socially isolated male rats have more CRF immuno-reactive cells expressed in the median eminence of the hypothalamus following an acute stressor compared to controls (Sánchez et al., 1998). Similarly, in prairie voles (*Microtus ochrogaster*), social stressors lead to an up-regulation of the CRF cell number and mRNA expressions in the PVN. Socially isolated females had more CRF immuno-reactive cells in the PVN after social defeat (Grippo et al., 2007), socially isolated males had higher CRF mRNA expression in the PVN compared to paired males (Pan et al., 2009), and in both genders social isolation led to higher density of CRF immuno-reactive cells in the PVN compared to voles paired with a con-specific (Ruscio et al., 2007). Similarly, isolation stress for at least 24 h and other stressors elevates CRF mRNA expression in the POA of rainbow trout (Doyon et al., 2005), and isolation for 24 h and 96 h lead to up-regulation of both CRF and UI mRNA expression in the POA of rainbow trout (Bernier et al., 2008). Thus, an activation of the PVN/POA CRF cell population is apparent after isolation stress. A similar up-regulation of CRF expression can be seen in dominance hierarchies as well. In a 14 days exposure to a visible burrow system in male rats (groups of five males and two females), subordinate males had higher CRF mRNA expression in the central amygdala compared to dominants and controls, and a subset of subordinate males (subordinate responders) had higher CRF mRNA expression in the PVN (Albeck et al., 1997). The subordinate responders were individuals responding with a higher corticosterone response compared to controls and dominants. However, 35 days of social defeat in tree shrews (*Tupaia belangeri*) also lead to fewer urocortin 1 immuno-reactive cells in the neuron population of the Edinger-Westphal nucleus and fewer CRF immuno-reactive cells in the parvocellular PVN and central amygdala (CeA) (Kozicz et al., 2008). Therefore, it seems that social defeat affects CRF expression in the PVN differently over time, and a similar pattern can be seen in teleost fish. In subordinate rainbow trout, CRF expression is up-regulated in the POA following 8 h interactions but not after 24 h interactions (Bernier et al., 2008). However, the time course for effects on CRF expression seems to be complex, since in another experiment rainbow trout being socially subordinate for 72 h show an up-regulation of CRF expression in the POA (Doyon et al., 2003). Further, after 5 days of social interactions, there were no differences in telencephalic or POA, CRF, or CRF-BP expression between dominant and subordinate rainbow trout (Jeffrey et al., 2012).

Differences between interacting individuals can also be seen in other regions of the brain. For instance, socially defeated male rats have lower CRF levels in the hippocampus (Panksepp et al., 2007). Further, in zebrafish (*Danio rerio*) the CRF mRNA levels are higher in the telencephalon of subordinate males (but not females) on day 1 of social interactions (Filby et al., 2010b). Similarly, CRF was up-regulated in the telencephalon of male subordinates, but CRF was also down-regulated in the hypothalamus of subordinate males and females following one day of social interactions (Filby et al., 2010a). However, after 5 days of social interactions, no difference in brain CRF mRNA could be seen in zebrafish (Filby et al., 2010b; Pavlidis et al., 2011). In *Astatotilapia burtoni* interacting for 4 weeks establishing territorial and non-territorial males, very similar to dominants and subordinates, respectively, whole brain CRF as well as pituitary CRF-R1 are down-regulated whereas CRF-BP is up-regulated in non-territorial males (Chen and Fernald, 2008). Further, visual contact for 3 days, but not 1 or 7 days, kept CRF, CRF-R2, and CRF-BP up-regulated whereas CRF-R1 was down-regulated in the brain of non-territorial males compared to controls (Chen and Fernald, 2011). Finally, Senegalese sole (*Solea senegalensis*) kept at high density for 33 days increase brain CRF mRNA expression as well as plasma cortisol levels compared to fish kept at low density, but no differences were seen in CRF-BP (Wunderink et al., 2011). All these differences are difficult to interpret, but are based on different brain parts. However, it is clear, as seen in zebrafish, rainbow trout, and *Astatotilapia burtoni*, that the effects of social interaction on CRF expression changes over time. This could be due to several different mechanisms, but aggressive behavior has been shown to be reduced over time in dyadic interacting zebrafish (Pavlidis et al., 2011), suggesting that social stress may decrease over time in interacting fish. Thus, the expression pattern of CRF in the brain is not universal and could be species-, context-, and time-dependent. It also seems that social stressors also sensitize the HPA axis. Icv injections of CRF into isolated rats lead to a higher increase of plasma corticosterone compared to controls (Serra et al., 2005). Similarly, rats socially defeated 2 days consecutively respond with more ACTH release after intravenous CRF injections after 7 days (Buwalda et al., 1999). However, this effect was not apparent at 21 days after social defeat (Buwalda et al., 1999). Thus, it seems as if the sensitization is only occurring during acute stressors and is probably down-regulated by the negative feedback system of the HPA axis.

The modulating effects of CRF are mediated by the CRF-receptors, which are responsible for separate responses. For instance, in socially defeated rats, injection with antisense CRF-R1 reduced anxiety-like behavior in the elevated plus maze, whereas antisense CRF-R2 increased immobility in forced swim test (Liebsch et al., 1999). Further, socially defeated CRF-R1 deficient mice had less impaired spatial memory compared to wild type mice (Wang et al., 2011), indicating that CRF-R1 promotes deleterious effects during social stress. In CRF-R2 knockout mice, aggression is increased, as indicated by a shorter latency to first attack and a higher number of attacks performed (Coste et al., 2006). Similarly, aggression in urocortin-2 knockout male mice is lower than in wild-type mice as measured by higher latency to first attack and more time in passive social contact (Breu et al., 2012). This indicates that CRF-R2 would be involved in the modulation of aggression. However, since socially isolated female prairie voles down-regulates CRF-R2 expression in the hypothalamus and up-regulates CRF-R2 expression in the hippocampus (Pournajafi-Nazarloo et al., 2009), and socially defeated rats seem to have up-regulated CRF-R2 mRNA in the posterior medial amygdala (Fekete et al., 2009), these effects could be site specific.

## Chronic effects of CRF—early life stress modulating adult behavior

Exposure to early life stress can have long-term effects on the development of neuroendocrine systems. These effects have been shown to increase risk for anxiety-like and depressive-like disorders in adulthood in humans [see Veenema (2009) and references therein]. Since CRF has been implied as being involved in anxiety-like behaviors, studies concerning its involvement in early life stress effects have been performed. One of the common models in studying early life stress is using the social stressor of maternal separation in different regimes. For instance, male rats suffering maternal separation and isolation through pre-adolescence and then re-socialized until early adulthood express several different anxiety-like behaviors, such as shorter duration of social interaction and longer duration of freezing (Lukkes et al., 2009a). Similarly, maternal separation for 3 h a day during pre-adolescence and then being re-socialized in groups of three induces the anxiety-like behaviors shown in elevated plus maze in male rats (Babygirija et al., 2012). The maternal separation and then re-socialization also induce a higher expression of CRF cells in the PVN as well as in the parvocellular division of PVN following a restraint stressor for 5 consecutive days (Babygirija et al., 2012), indicating a more sensitized HPA axis in these rats. However, most of these effects could be ameliorated if the animal is re-socialized with naïve rats. Further, in male prairie voles, social isolation for 6 weeks post-weaning increased anxiety-like behavior in elevated plus maze and increased CRF mRNA levels in the PVN (Pan et al., 2009). Thus, it seems that early life stress increases anxiety-like behaviors as well as sensitizes the HPA axis in adulthood. The anxiety-like behavior seems to be controlled by the CRF-R2. For instance, in male rats going through the maternal separation and then re-socialization, the anxiety-like behavior is reduced by injection of a general CRF antagonist (D-Phe-CRF_(12−41)_) into the dorsal raphe nucleus (Lukkes et al., 2009a) and the CRF-R2 expression is up-regulated in the dorsal raphe nucleus (Lukkes et al., 2009b). Further studies showed that the specific CRF-R1 antagonist antalarmin and the specific CRF-R2 antagonist anti-sauvagine injected into the dorsal raphe nucleus both modulated the induced anxiety-like behaviors. However, anti-sauvagine reversed all anxiety-like behavior whereas antalarmin only had minor effects (Bledsoe et al., 2011). These studies all indicate that CRF-R2 in the dorsal raphe nucleus is involved in the anxiety-like behavior modulation following early life stress. However, in a study using wild-type and CRF-R2 knockout mice in a context fear memory study, maternal separation and isolation post-weaning induced more fear responses in wild-type and knockouts as compared to controls reared in groups of three (Gresack et al., 2010), thus indicating other pathways inducing fear and anxiety.

## Strain differences—the impact of CRF on stress coping styles

In mammals, divergent stress responses can be consistent over context and time. The behavioral and physiological responses to stress can generally be divided into two coping styles, namely proactive and reactive stress coping (Koolhaas et al., 1999, 2007). Proactive animals are more active, behave more aggressively, and readily form behavioral routines as compared to reactive animals (see Table 1). Moreover, proactive animals show lower HPA axis reactivity but higher sympathetic reactivity than reactive animals. Since divergent coping styles appear related to differences HPA-axis reactivity and locomotory activity, which are both under the control of CRF [see reviews by Heinrichs and Koob (2004) and Lowry and Moore (2006)], differences in the CRF system should be apparent.

**Table 1:** **Summary of the behavioral and physiological differences between proactive and reactive animals**.

	**Proactive**	**Reactive**
**BEHAVIORAL CHARACTERISTICS**		
Aggression	High	Low
Conditioned immobility	Low	High
Routine formation	High	Low
**PHYSIOLOGICAL CHARACTERISTICS**		
HPA axis reactivity	Low	High
Parasympathetic reactivity	Low	High
Sympathetic reactivity	High	Low

Modified from Koolhaas et al. (1999, 2007) and Øverli et al. (2007) and references therein.

Few studies in mammals have addressed this possible link. However, rodents with similar differences in stress responsiveness have been studied. For instance, rats bred for high anxiety behavior (HAB) or low anxiety behavior (LAB) also seem to differ in stress coping style (Landgraf and Wigger, 2002). The HAB rats seem to be more passive and have a higher HPA axis reactivity, thus fitting nicely into the reactive stress coping style. Interestingly, there seems to be differences in the CRF system between HAB and LAB rats. During basal conditions, HAB rats display lower CRF mRNA in the bed nucleus of the stria terminalis, but higher CRF-R2 expression in the PVN and the ventromedial hypothalamus than LAB rats (Wigger et al., 2004). Following stress, HAB rats show higher CRF-R2 expression in the ventromedial hypothalamus and the central amygdala (Wigger et al., 2004) as compared to LAB rats. Thus, differences in stress coping style seem to be reflected in differences in the CRF system. Similarly, in rats subjected to social defeat for 7 days, the behavioral reactivity could be divided into short latency (SL) or long latency (LL) to assume submissive posture (Wood et al., 2010). These two groups also fit nicely with reactive and proactive stress coping styles, respectively. CRF mRNA densities in the PVN and CRF-R1 levels in the pituitary were decreased in the SL rats compared to controls 24 h after last social defeat (Wood et al., 2010). Further, in mice strains selected for aggressiveness based on long attack latency (LAL) or short attack latency (SAL), no difference in CRF mRNA in PVN was noted during basal conditions, but 24 h after a swim stress LAL mice had higher CRF expression in the PVN than basal LAL and stressed SAL (Veenema et al., 2003). Socially defeated mice could be divided into active (proactive) or passive (reactive) coping, and the active coping mice had higher CRF mRNA levels in the hypothalamus than passive or control mice 1 h after social stress (De Miguel et al., 2011). Several of these differences in the CRF system also make sense concerning the stress coping styles, although differences once again are dependent on time. The reactive stress coping style having a more sensitized CRF system, including elevated CRF and CRF-R1 expression in the PVN during acute social stressor, leading to a higher HPA-axis reactivity. Similarly, high CRF expression in mice reduces aggression.

In recent years, studies on teleost fish have established the existence of stress coping styles in several different species such as halibut (*Hippoglossus hippoglossus*) (Kristiansen and Fernö, 2007), Nile tilapia (*Oreochromis niloticus*) (Barreto and Volpato, 2011), and Senegalese sole (Silva et al., 2010). Further, salmonid species have been examined thoroughly. For instance, in brown trout (*Salmo trutta*) individuals clustered into two separate stress coping styles based on the plasma levels of noradrenaline and adrenaline post-confinement and behavior during hypoxia (Brelin et al., 2005). Similarly, in rainbow trout aggression, dominance and post-stress plasma levels of cortisol differed between individuals and resulted in two distinct coping styles (Schjolden et al., 2005b). Further, two selected strains of rainbow trout representing high responders (HR) and low responders (LR) with respect to plasma cortisol concentrations following a standardized confinement stress were studied by Pottinger and Carrick (1999). Over a series of experiments, these strains were shown to correspond to reactive and proactive stress coping strategy, respectively [see review by Øverli et al. (2007)]. A recent study presented results suggesting divergent effects of stress on the CRF system in HR and LR trout (Backström et al., 2011b). HR trout subjected to confinement stress for 180 min showed higher CRF mRNA levels than LR trout exposed to the same stressor, and following a 30 min confinement HR trout displayed higher CRF-R1 and lower CRF-R2 mRNA levels than LR trout (Backström et al., 2011b). Most likely, these differences are related to the divergent stress coping styles expressed by HR and LR trout. For instance, CRF has been reported to reduce feed intake in teleost fish (De Pedro et al., 1993; Bernier and Peter, 2001), and following a stressful challenge LR fish regain feed intake faster than the HR fish (Øverli et al., 2002). Thus, the higher expression of CRF mRNA in the HR strain during stress could mediate an anorexic effect in the HR strain. Further, CRF has been reported to increase locomotor activity in teleost fish (Clements et al., 2002; Clements and Schreck, 2004). Previous reports have shown diverging activity between the HR and LR fish. The HR fish has been reported to have lower or higher activity than the LR fish (Øverli et al., 2002; Schjolden et al., 2005a, 2006; Backström et al., 2011b). These effects appear to be inconsistent and could be context based. It appears as if LR fish show higher activity during non-stressful, whereas HR fish show higher activity during stressful conditions. The HR fish displayed lower activity when kept in groups in large tanks, in open field, and during isolation (Øverli et al., 2002; Schjolden et al., 2005a, 2006) but showed higher activity during resident-intruder tests (Øverli et al., 2002) and when in confinement (Backström et al., 2011b). This divergence between strains concerning activity could be due to the differences in the stress-induced effects on CRF release. Furthermore, CRF is involved in the control of aggressive behavior and has been reported to suppress aggressive behavior in rainbow trout (Backström et al., 2011a). This means that the diverging neuroendocrine stress responses between strains could explain strain related differences in aggression. The HR strain has been proposed to be less aggressive. For instance, Pottinger and Carrick (2001) reported that when interacting in size matched pairs, HR fish became subordinate significantly more often than LR fish. In addition, the higher locomotor activity shown by LR fish when reared in larger groups was suggested to be caused by high levels of agonistic interaction (Schjolden et al., 2006).

## Concluding remarks

CRF and its related peptides are involved in several behavioral and physiological responses to social stressors. These modulating effects are likely to be mediated through several different mechanisms and neuronal structures. The PVN/POA is involved in the HPA/HPI axis regulation through the CRF-R1. Both CRF-R1 and CRF-R2 seems to modulate the behavioral responses to social stress, such as aggression and anxiety-like behavior. Specifically, CRF-R1 seems to be more directly involved in modulating anxiety-like behavior, whereas CRF-R2 has more pronounced effects on aggressive behavior. However, some studies have also seen modulating effects of CRF-R1 on aggressive behavior. The effects of early life stress on stress responsiveness and anxiety-like behavior in adulthood appear to be at least in part mediated by CRF-R2 expressed in the dorsal raphe nucleus. Finally, differences in the CRF system is probably one factor responsible for the differences observed in behavioral profiles and stress responses of individuals displaying divergent stress coping styles. Since most of these studies have been performed on juvenile teleost fish and male rodents, further studies are needed to elucidate if there are also gender differences in the CRF system. Furthermore, the links between CRF and several other pathways including the vasopressinergic and monoaminergic systems are needed for a better understanding of the effects of social stress on behavior and physiology.

### Conflict of interest statement

The authors declare that the research was conducted in the absence of any commercial or financial relationships that could be construed as a potential conflict of interest.
